# Supplementary material for: The Impact of Medication Regimen Adjustment Ratio on Adherence and Glycemic Control in Patients with Type 2 Diabetes and Mild Cognitive Impairment
Source: Biomedicines. 2024 Sep 16;12(9):2110. doi: 10.3390/biomedicines12092110 (PMC11429028; doi:10.3390/biomedicines12092110)
Supplement: Supplementary file 1 [file biomedicines-12-02110-s001.zip › biomedicines-3180394-supplementary.pdf]

Supplemental Table S1. Subgroup analysis of a single anti-diabetic drug in MCI patients (*n*=35)

**Characteristics**	**Adjustment ratio of anti-diabetic drugs**		***P***
	**0%**	**100%**	
Baseline			
Age (years)	54.0 (49.5,58.5)	58.5 (52.7,62.5)	0.188
Diabetes duration (years)	10.0 (7.5,12.5)	7.5 (0.0,15.5)	0.321
HbA1c%	6.3 (5.9,9.6)	8.3 (7.5,9.3)	0.030
MoCA	23.0 (22.0,24.5)	22.0 (20.0,23.0)	0.159
MMSE	28.0 (26.0,29.0)	26.0 (25.0,28.0)	0.038
Low education	10 (76.9)	20 (90.9)	0.253
MARS-5, ≥23	13 (100)	21 (95.5)	1.000
<23	0	1 (4.5)	
3-month follow-up			
ΔHbA1c (%)	0.2 (-0.2,0.3)	0.3 (-0.4,0.6)	0.410
ΔMoCA	1.0 (0.5,2.0)	1.5 (-2.0,2.0)	0.844
ΔMMSE	0 (0,0.0)	0 (-0.2,0.0)	0.192
MARS-5, ≥23	11 (84.6)	14 (63.6)	0.259
<23	2 (15.4)	8 (36.4)	

Data are expressed as median (quartile) or *n* (%). MCI—mild cognitive impairment; HbA1c—hemoglobin A1c; MoCA—Montreal Cognitive Assessment; MMSE—Mini-mental State Examination; MARS-5—the Medication Adherence Report Scale-5; ΔHbA1c—$HbA1c_{3\text{-month}}-HbA1c_{baseline}$; ΔMoCA—$MoCA_{3\text{-month}}-MoCA_{baseline}$; ΔMMSE—$MMSE_{3\text{-month}}-MMSE_{baseline}$.

Supplemental Table S2. Subgroup analysis of two or three oral anti-diabetic drugs in MCI patients (*n*=24)

**Characteristics**	**Adjustment ratio of anti-diabetic drugs**		***P***
	**<50%**	**≥50%**	
Baseline			
Age (years)	62.0 (55.0,66.5)	53.0 (51.0,62.00	0.155
Diabetes duration (years)	6.0 (3.0,9.0)	7.0 (3.0, 10.0)	0.560
HbA1c%	6.9±1.3	7.5±2.2	0.408
MoCA	21.3±2.1	22.1±1.9	0.349
MMSE	26.9±1.7	27.5±1.7	0.454
Low education	10 (76.9)	9 (81.8)	0.769
MARS-5, ≥23	12 (92.3)	11(100.0)	0.347
<23	1 (7.7)	0	
3-month follow-up			
ΔHbA1c%	-0.1±0.5	-0.1±0.5	0.824
ΔMoCA	1.0 (0.0,2.0)	2.0 (2.0,2.0)	0.035
ΔMMSE	0 (0.0,0.5)	0 (0.0,0.0)	1.000
MARS-5, ≥23	10 (76.9)	10 (90.0)	0.360
<23	3 (23.1)	1 (9.1)	

Data are expressed as median (quartile) or *n* (%). MCI—mild cognitive impairment; HbA1c—hemoglobin A1c; MoCA—Montreal Cognitive Assessment; MMSE—Mini-mental State Examination; MARS-5—the Medication Adherence Report Scale-5; ΔHbA1c—$HbA1c_{3\text{-month}}-HbA1c_{baseline}$; ΔMoCA—$MoCA_{3\text{-month}}-MoCA_{baseline}$; ΔMMSE—$MMSE_{3\text{-month}}-MMSE_{baseline}$.
